# Microenvironment-responsive peptide hydrogels: molecular mechanisms, design and frontiers

**DOI:** 10.3389/fbioe.2025.1692319

**Published:** 2025-11-13

**Authors:** Xi-kun Ma, Biao Cao, Xi Liu

**Affiliations:** 1 Department of Orthopedic Surgery and Orthopedic Research Institute, West China Hospital, Sichuan University, Chengdu, Sichuan, China; 2 Department of Orthopaedic Surgery, Shangjin Nanfu Hospital, Chengdu, Sichuan, China

**Keywords:** peptide hydrogels, microenvironment-responsive, wound healing, drug delivery, smart biomaterials

## Abstract

Responsive peptide hydrogels are advanced platforms for wound management because they can dynamically interact with the wound microenvironment. These smart materials respond to specific biochemical cues such as pH, reactive oxygen species (ROS), matrix metalloproteinases (MMPs), and glucose (Glu), enabling precise control over drug release, enhancement of cellular repair, and suppression of infection. By adapting to pathological conditions like elevated pH, persistent oxidative stress, and enzymatic imbalances, peptide hydrogels promote efficient healing in chronic wounds. Recent advances have expanded their responsiveness to include physical stimuli like temperature, light, and magnetic fields, broadening their applicability in deep and complex wound treatments. Despite promising outcomes, challenges remain in optimizing biocompatibility, biodegradability, and stimulus precision. Future efforts will focus on developing multifunctional and personalized hydrogel systems to achieve smarter, minimally invasive therapeutic strategies for wound care and beyond.

## Highlights

- Comprehensive review of microenvironment-responsive peptide hydrogels, detailing their molecular mechanisms in response to key pathological factors (pH, ROS, MMPs, Glu) and physical stimuli (temperature, light, magnetism) for advanced wound management.
- Emphasis on clinical translation potential, highlighting applications in chronic and diabetic wound healing through intelligent drug release, antimicrobial action, and promotion of cellular repair processes.
- Discussion on future frontiers and challenges, addressing the need for improved biocompatibility, multi-functionality, and personalized hydrogel systems to achieve smarter therapeutic strategies.

## 1 Introduction

Wound healing occurs through four distinct stages: coagulation, hemostasis, inflammation, proliferation, and remodeling (Liang et al., 2021; Liang et al., 2022). When these processes fail to be properly coordinated, the wound progresses from an acute to a chronic state. Acute wounds typically achieve clinical healing through standard management protocols. However, with the global increase in the aging population and the rising prevalence of diabetes, the burden of managing chronic wounds is escalating (Tehrany et al., 2023). Furthermore, the growing issue of bacterial resistance complicates wound healing, creating significant challenges for healthcare systems worldwide (Castaño et al., 2018).

**FIGURE 1:**
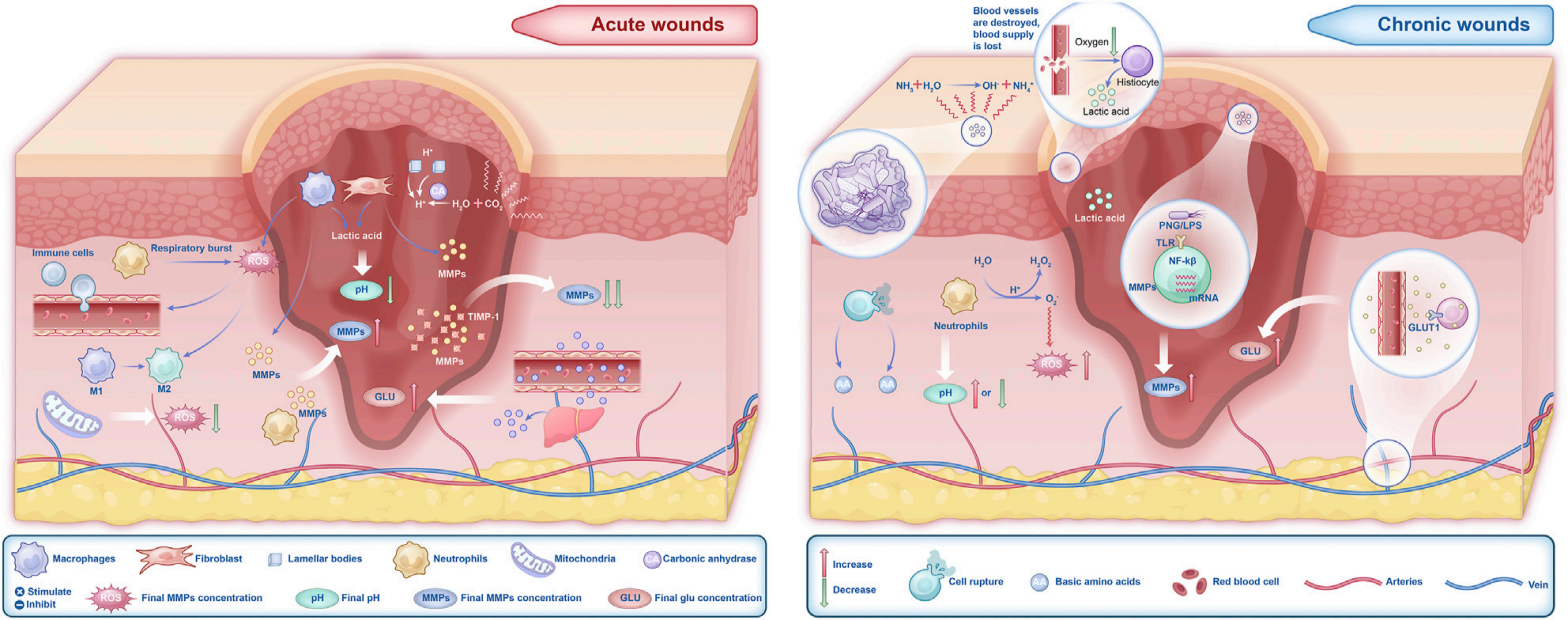
Microenvironmental differences between acute and chronic wounds.

**FIGURE 2:**
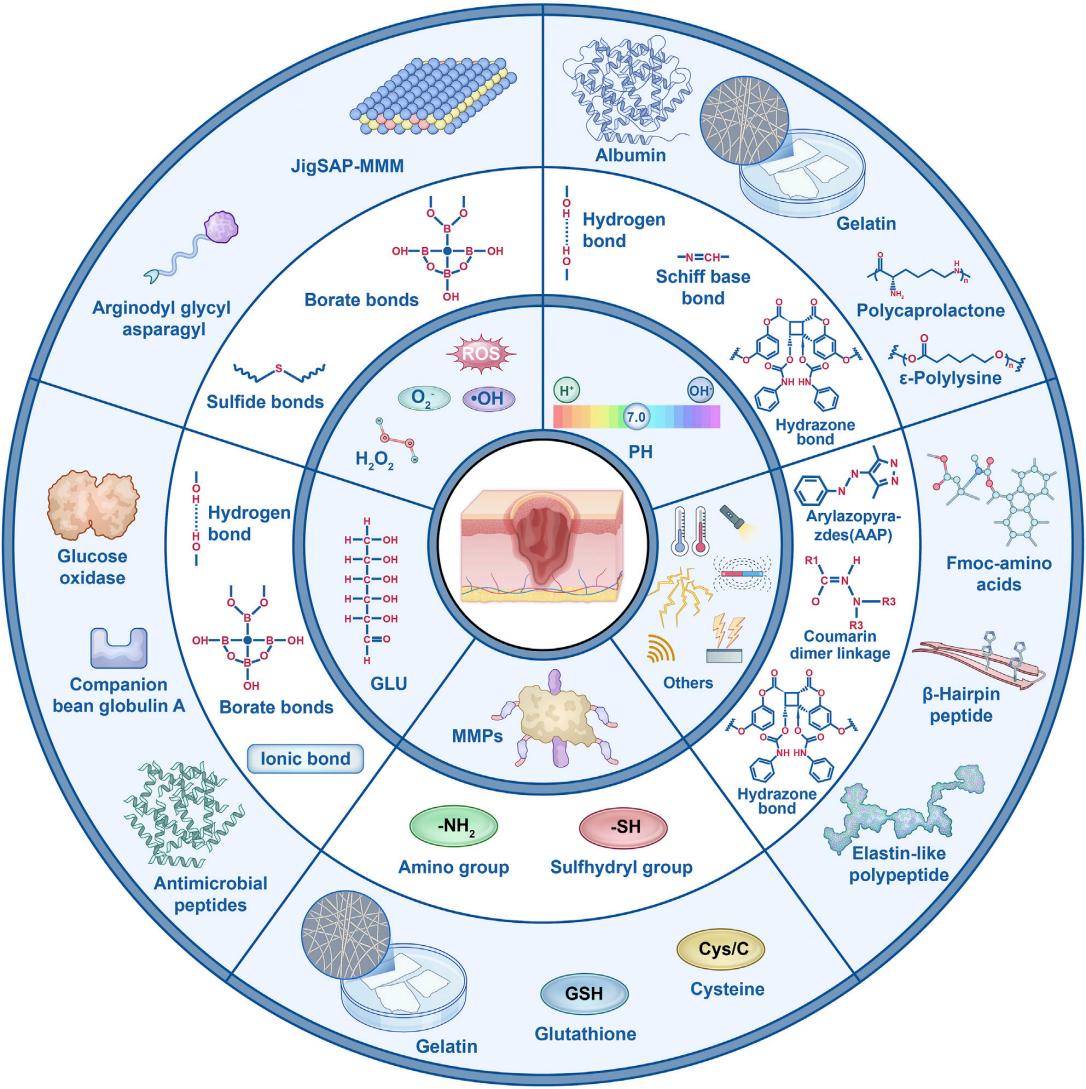
Key functional groups of various responsive peptide hydrogels and their representative applications.

Regardless of whether the wound is acute or chronic, changes in the microenvironment significantly influence the healing process (Kirchner et al., 2020), particularly the components such as pH, ROS, MMPs, and Glu levels (Figure 1). The microenvironment of acute wounds generally exhibits a lower pH. This acidity stems primarily from the glycolytic activity of macrophages and the Warburg effect in fibroblasts, processes that increase lactic acid production and consequently lower the pH (Barker et al., 2017; Mahanty, 2025). In contrast, chronic wounds typically have a higher pH, ranging from 7.3 to 8.9 (Wilson et al., 1979), due to bacterial biofilm formation, ischemia, and hypoxia. However, prolonged bacterial infections may result in a decline in wound pH. ROS levels are elevated in the early stages of acute wounds (Zhang et al., 2023a; Suzuki et al., 2011), where they effectively inhibit bacterial growth (Rodrigues et al., 2019). In chronic wounds, however, ROS levels remain persistently high. This sustained oxidative stress impairs critical healing processes, including macrophage transformation, collagen synthesis by fibroblasts, and angiogenesis, thereby delaying wound healing (Zhao et al., 2023; Xue et al., 2024; Deng et al., 2019; Li et al., 2021; Janda et al., 2016; Gouzos et al., 2020). Additionally, excessive ROS activity leads to the sustained secretion of MMPs, exacerbating inflammation and tissue degradation (Raziyeva et al., 2021; Daraban Bocaneti et al., 2022; Xing et al., 2017). Fluctuations in Glu levels also play a crucial role in wound healing. Elevated Glu levels in both acute and chronic wounds provide cellular energy, but they also increase the risk of infection and suppress the release of angiogenic factors, leading to impaired proliferation during the healing process (Dam and Paller, 2018). Therefore, changes in the wound microenvironment play a critical and undeniable role in the healing trajectory of wounds (Figure 2).

In recent years, hydrogels have emerged as an ideal wound dressing due to their excellent biocompatibility, moisture retention, and transparency, demonstrating substantial potential for development (Chen et al., 2023; Khattak et al., 2024; Khattak et al., 2025b; Khattak et al., 2025a; Zheng et al., 2024). Hydrogels not only maintain wound moisture but also serve as drug delivery vehicles, enabling the precise delivery of therapeutic agents to targeted areas and effectively modulating the wound microenvironment (Merino et al., 2015; Liu et al., 2019). With the advancement of biomaterials research, responsive hydrogels, also known as smart hydrogels, have become a focal point of study (Li and Su, 2018). Responsive peptide hydrogels, composed of short-chain amino acids, self-assemble into bioactive nanostructures through non-covalent interactions, such as hydrogen bonding and π-π stacking (Zhou et al., 2024). Their controllable self-assembly properties and significant bioactivity enable them to dynamically respond to key changes in the chronic wound microenvironment—such as pH, ROS, and enzymatic activity—thereby aligning with the specific pathophysiology of chronic wounds (Sheehan et al., 2021). Consequently, responsive peptide hydrogels, through precise matching with the wound microenvironment, can promote the healing of chronic wounds while preventing prolonged inflammation and tissue damage. As hydrogel materials continue to evolve, they are poised to demonstrate broader application potential in the treatment of chronic wounds (Tao et al., 2017; Huo et al., 2023; Bera et al., 2019). Beyond their significant advantages in chronic wound healing, this class of hydrogels demonstrates broad application prospects in various disease areas. In cancer therapy, pH- or MMP-responsive peptide hydrogels can target the tumor microenvironment to enable localized release of anticancer drugs enhancing therapeutic efficacy while reducing systemic toxicity. For instance, hydrogels containing thioether or boronic ester bonds can trigger drug release in tumors with ROS overexpression and have also been utilized for tumor imaging (Zha et al., 2021). In the context of central nervous system diseases, MMP-responsive hydrogels have been applied for neuroprotection or neuroregeneration after stroke, releasing neurotrophic factors in response to the local inflammatory microenvironment. Furthermore, these hydrogels have shown important progress in bone and cartilage repair (Li et al., 2022; Zhang et al., 2024a; Ren et al., 2020), cardiovascular diseases (Yang et al., 2022), and inflammatory skin diseases (Noddeland et al., 2023). Microenvironment-responsive peptide hydrogels, with their high biocompatibility, programmable smart-response mechanisms, and multifunctional integration capabilities, are gradually emerging as a promising platform technology for precision therapy and tissue engineering in a variety of diseases.

Given the pivotal role of the wound microenvironment in healing outcomes and the unique potential of hydrogels to modulate it, this review aims to provide a timely and comprehensive analysis of microenvironment-responsive peptide hydrogels, a class of smart materials designed to interact dynamically with pathological cues. We will systematically elucidate the molecular mechanisms and design principles of hydrogels engineered to respond to key biochemical stimuli in the wound bed. The review will focus specifically on pH-responsive, ROS-scavenging, MMP-degradable, and Glu -sensitive peptide hydrogels, examining their capabilities in promoting targeted drug delivery and tissue regeneration. Furthermore, we will explore the emerging frontier of hydrogels responsive to physical stimuli, such as temperature and light, and discuss the current challenges and future directions for translating these advanced biomaterials into clinical practice.

## 2 Microenvironmentally stimuli-responsive peptide hydrogels

### 2.1 pH-responsive peptide hydrogels

The application of pH-responsive hydrogels in wound healing is particularly significant, as the pH of wound exudates can vary considerably depending on factors such as wound type, healing stage, and infection. Based on this characteristic, pH-responsive hydrogels are expected to become an ideal choice for wound dressings. The pH-responsive behavior of hydrogels primarily arises from the ionizable side groups in the polymer backbone (Gupta et al., 2002). When exposed to an appropriate pH and ionic strength, these side groups ionize and accumulate charges, generating electrostatic repulsion that causes the hydrogel to swell or deswell (Sharpe et al., 2014; Peppas et al., 2000). Depending on the ionization and swelling behaviors, pH-responsive hydrogels can be categorized into two types. Anionic hydrogels remain collapsed at low pH, whereas an increase in pH leads to ionization-induced electrostatic repulsion and water absorption, causing the hydrogel to swell. Conversely, cationic hydrogels exhibit the opposite behavior. Common monomers used to introduce pH responsiveness include acrylic acid (AA), methacrylic acid (MAA), and acrylamide (AAm) (Koetting et al., 2015). Natural polymers such as albumin, gelatin (Welz and Ofner, 1992), alginate, and chitosan also demonstrate pH responsiveness. For example, albumin and gelatin can form stable helical structures under specific pH and temperature conditions. These structures act as crosslinking points, thereby modulating the hydrogel’s swelling behavior. Chitosan and alginate undergo physical crosslinking through charge or hydrophobic interactions, expanding upon ionization, leading to charge accumulation and electrostatic repulsion.

Natural pH-responsive polymers possess excellent biodegradability, making them particularly suitable for *in vivo* applications, especially in drug delivery and wound healing (Schmaljohann, 2006). In addition to these polymers, reversible chemical bonds such as Schiff bases can also be used to modulate the structure and physicochemical properties of hydrogels (Guo et al., 2022; Sacks et al., 2018). By combining antimicrobial peptides (AMPs) with pH-responsive hydrogels, drugs can be precisely released in response to changes in wound pH, enhancing antimicrobial efficacy. Some studies have incorporated antimicrobial tetrapeptides into polycaprolactone (PCL) embedded in sodium alginate (SA) and N-carboxymethyl chitosan (NCMC) hydrogels, using NCMC to control the release in the neutral/alkaline liquid environment of wounds (Miranda et al., 2023). For diabetic wound infections, one study developed a bifunctional pH-sensitive hydrogel based on the cationic antimicrobial peptide DP7 and oxidized dextran. This hydrogel can simultaneously load antibiotics and AMPs to exert synergistic antimicrobial effects, demonstrating substantial therapeutic potentiall (Zhang et al., 2023c; Wu et al., 2022b).

In addition, many pH-responsive hydrogels utilize chitosan and its derivatives, acrylic acid and its derivatives, as well as carboxymethyl agarose derivatives as substrates. These materials are often combined with multifunctional components such as chondroitin sulfate, tannic acid, metal ions, and plant extracts (e.g., quercetin), and have been widely applied in areas such as controlling wound infection and promoting healing (Wang et al., 2022a; Haidari et al., 2022; Wu et al., 2022a; Resina et al., 2023).

### 2.2 ROS-responsive peptide hydrogels

ROS are highly reactive ions generated in the human body as byproducts of aerobic respiration, including hydrogen peroxide, superoxide anions, and hydroxyl radicals (Tyagi et al., 2021). While ROS play an essential role in wound healing, their dual nature cannot be overlooked. In the early stages of healing, ROS contribute to infection control by eliminating pathogens. In the later stages, low concentrations of ROS stimulate the polarization of M2 macrophages, promoting tissue repair. However, excessive ROS levels can lead to prolonged inflammation and degradation of the extracellular matrix (ECM). This imbalance drives the wound into a chronic, non-healing state (Dunnill et al., 2017). Currently, ROS-responsive hydrogels can be broadly classified into two types. The first type involves the degradation of hydrogels in oxidative environments, altering their properties (e.g., drug release or swelling characteristics). Common responsive units in these systems include thioketal bonds, diselenide bonds, and boronate ester bonds. The second type of ROS-responsive hydrogels changes the solubility of the material (from hydrophobic to hydrophilic) in response to ROS oxidation, achieving the desired effect. These hydrogels typically contain responsive units such as thioether bonds and ferrocene (Saravanakumar et al., 2017).

Among these, boronate ester bonds are the most widely applied. Under the presence of ROS, boronate ester bonds undergo oxidative cleavage, releasing active components and eliminating ROS. They exhibit temperature and pH responsiveness and are commonly found in multi-responsive hydrogel systems. As proposed by Pengfei Wang in his study, the phenylboronic acid moiety is one of the most frequently used ROS-triggered groups for designing ROS-responsive prodrugs. It enhances the lipophilicity of the drug, thereby increasing its therapeutic efficacy and providing more sustained drug activity for wound healing (Wang et al., 2021). Despite the tremendous potential of ROS-responsive materials in biomedicine, challenges remain in their application. These include uncertainties regarding their behavior under different physiological conditions, degradation products, and the lack of extensive *in vitro* toxicity and *in vivo* studies (Yao et al., 2019). Overall, ROS-responsive biomaterials offer promising strategies for biomedical treatments but require further investigation to address these challenges.

### 2.3 MMP-responsive peptide hydrogel

The activity and expression of MMPs in the human body are strictly regulated under physiological conditions by tissue inhibitors of metalloproteinases (TIMPs), cytokines, hormones, and cell-to-cell interactions. In healthy tissues, MMP levels are low and their activity is limited. However, in pathological conditions such as inflammatory skin diseases and chronic wounds, MMP activity is significantly upregulated (Noddeland et al., 2023). The mechanism of MMP-responsive hydrogel systems is primarily based on proteins or peptides that can be hydrolyzed by MMPs. When exposed to an environment with sufficient MMP concentrations, these peptides act as substrates, undergoing catalytic reactions that result in the degradation of the hydrogel or drug-loaded microparticles, thus releasing the encapsulated drug (Lei and Segura, 2009). Currently, two common approaches for introducing MMP-responsive behavior into hydrogels are: first, using gelatin as an MMP substrate (which can serve as a matrix or as a carrier for encapsulating drugs or RNA); and second, incorporating MMP-sensitive peptide side chains into the hydrogel matrix or as crosslinking agents.

Gelatin, a natural protein derived from animal connective tissues, is a recognized MMP substrate and is widely used in hydrogel matrices (Fan et al., 2022), microspheres (Cai et al., 2022; Liu et al., 2018; Shao et al., 2023), and nanoparticles (Zhang et al., 2023b). These applications span across wound dressings, tissue regeneration, and vascular reconstruction. For instance, For instance, Ribeiro et al. developed an injectable gelatin methacryloyl (GelMA) hydrogel modified with nanotubes loaded with chlorhexidine (CHX). This system serves as an injectable drug delivery platform for clinical infection ablation and has demonstrated excellent antimicrobial efficacy (Ribeiro et al., 2020). Gelatin offers outstanding stability and biocompatibility, and its applications include hydrogel matrices, microspheres encapsulating drugs or RNA, and gelatin nanoparticles as drug carriers. However, there are still limitations in the development of gelatin, particularly in the field of hydrogel matrices, where research remains insufficient and its application scope is relatively narrow. Future studies may explore new drug delivery methods to expand its potential.

Additionally, MMP-sensitive peptides are commonly used to introduce MMP-responsive behavior. Typical applications include using MMP-sensitive peptides as crosslinking agents, as modifiers of the hydrogel matrix, or directly within the matrix itself. Among these, the most common approach is to use MMP-sensitive peptides as crosslinkers to form responsive hydrogel matrices, which can then be used to load drugs or other components. This approach has been combined with materials such as polyethylene glycol (PEG), hyaluronic acid, collagen, and functionalized chitosan, and has been widely applied in promoting wound healing. For example, Daviran et al. designed a hydrogel loaded with human mesenchymal stem cells (hMSC) related to wound healing, where the hydrogel was chemically crosslinked from PEG and MMP-degradable peptide sequences, enabling the release of hMSCs to promote wound healing (Daviran et al., 2020). Thai et al. developed three-dimensional cell spheroids capable of secreting elevated levels of endogenous nutrients, a key factor for promoting cell proliferation. They used an MMP-sensitive crosslinker to form PEG hydrogels as a scaffold for the cell aggregates, and the hydrogel degraded progressively as the secreted MMPs increased, perfectly matching the requirements for enhanced cellular vitality (Thai et al., 2023). This indicates that responsive peptide hydrogels can achieve dynamic responses and precise matching during the healing and restoration of chronic wounds.

### 2.4 Glu-responsive peptide hydrogels

The high levels of Glu in the wound microenvironment, particularly in diabetic patients, are a major cause of wound infection and a significant obstacle to wound healing. In this context, Glu-responsive hydrogels, used as wound dressings, can regulate drug release based on the hyperglycemic environment (Chen et al., 2023). These hydrogels achieve responsiveness primarily through three mechanisms: the phenylboronic acid (PBA) dynamic covalent bond system, enzyme-catalyzed cascade reaction system, and lectin-specific binding system.

In the PBA dynamic covalent bond system, PBA forms a Glu-responsive hydrogel by interacting with hyaluronic acid methacrylate (HAMA) (Xu et al., 2022). Upon reaction with Glu, the hydroxyl groups of PBA form reversible boronic ester bonds, which leads to the release of the loaded drug. Additionally, these hydrogels exhibit antioxidant properties (Xu et al., 2022), capable of scavenging ROS and protecting cells from oxidative stress-induced damage. Studies (Xu et al., 2022) have demonstrated that when combined with natural polyphenol catechins (Gao et al., 2021; Chen et al., 2020), the hydrogel promotes angiogenesis (increased expression of VEGF and CD_31_) and reduces inflammation (lower IL-6 levels and increased IL-10 levels), thereby accelerating wound healing (Xu et al., 2022).

In the enzyme-catalyzed cascade reaction system, glucose oxidase (GO_x_) is employed to modulate the high- Glu environment of the wound. GO_x_ catalyzes the conversion of Glu to gluconic acid and hydrogen peroxide (H_2_O_2_) (Sacks et al., 2018), triggering a cascade reaction: gluconic acid lowers the local pH, which breaks pH-sensitive bonds (such as imine bonds), while H_2_O_2_ activates ROS-responsive elements (such as thioether bonds). To enhance the functionality of GOx, which inherently lacks additional biological activities, the team led by Yuheng Liao developed Au-FePS3 nanosheets by immobilizing GOx-loaded gold nanoparticles onto FePS3 nanosheets.This system not only preserves the cascade reaction characteristics but also exhibits antibacterial properties, promotes oxygenation, and stimulates endothelial cell proliferation (Jovin et al., 2015; Kennedy et al., 2016; Kidwell et al., 2013; Mocco et al., 2016).

The lectin-specific binding system works by crosslinking lectins (such as Concanavalin A, ConA) with polymeric sugar chains to form a network (Goldstein and Hayes, 1978; Gabor et al., 2004). Under high Glu conditions, Glu competes with lectins for binding, causing the hydrogel network to dissociate and release the loaded drug. ConA, known for its high affinity and reversible binding, is an ideal choice as it can bind with Glu to induce hydrogel swelling and regulate the high-Glu environment of the wound (Wang et al., 2019; Brownlee and Cerami, 1979; Seo Young et al., 1984). However, the volatility of ConA necessitates its effective immobilization. The team led by Zhang et al. (2006) successfully stabilized ConA by chemical modifications (carbodiimide, epoxy ring-opening reactions, and Schiff base reactions), thereby enhancing its stability (Pal et al., 2025).

In summary, Glu-responsive hydrogels precisely regulate drug release through multiple mechanisms, addressing the challenges of a hyperglycemic environment while also exhibiting antioxidant, antibacterial, and wound-healing-promoting functions. These hydrogels represent an emerging and effective therapeutic strategy for wound treatment in diabetic patients and those with other high-Glu conditions.

### 2.5 Other stimuli-responsive peptide hydrogels

In recent years, responsive peptide hydrogels designed for chronic wound repair have experienced rapid growth, particularly in the past 3–5 years. In addition to traditional peptide hydrogels that respond to changes in the wound microenvironment, such as pH, ROS, MMPs, and Glu, hydrogels responsive to other stimuli, such as temperature (Chi et al., 2020; Pal et al., 2020; Zhang et al., 2021; Chen et al., 2022), ultrasound (Chen et al., 2022), electric fields, pressure, magnetic fields (Wang et al., 2022b), infrared (IR), ultraviolet (UV), and photothermal effects, have also been developed (Su et al., 2023). Temperature, a common stimulus, can be categorized into low-temperature and high-temperature stimuli. Under low-temperature conditions, hydrogels exhibit positive responsiveness (Upper Critical Solution Temperature, UCST), while high-temperature stimulation results in negative responsiveness (Lower Critical Solution Temperature, LCST) (Dzuricky et al., 2018). Temperature fluctuations induce changes in the hydrogel state and mechanical properties (Chatterjee and Hui, 2021), with 37 °C, the body’s constant temperature, serving as a key control point for many temperature-responsive hydrogels (Chatterjee and Hui, 2021). For example, poly (N-isopropylacrylamide) (PNIPAM) hydrogels exhibit an LCST near body temperature, at which point the hydrogel undergoes swelling and contraction (Cheng et al., 2018). The Fang team has leveraged temperature-responsive hydrogels for targeted cell therapy, thereby promoting homeostasis and repair (Fang et al., 2018). Consequently, temperature-responsive hydrogels with an LCST close to or below 37 °C hold great potential for applications in wound healing, providing a precise trigger mechanism without the need for external interventions (Zhang et al., 2024b).

Light stimulation is a non-invasive, spatiotemporally controllable, and energy-adjustable stimulus widely used in biomedical applications (Zhou et al., 2024). Light-responsive peptide hydrogels incorporate photosensitive groups, such as azobenzene (Zhou et al., 2024) or coumarin, into the peptide structure. Upon exposure to specific wavelengths (UV, visible, or near-infrared light), these hydrogels undergo photochemical reactions that alter their molecular conformation, hydrophilicity/hydrophobicity, and crosslinking density, thereby controlling drug release and modulating the microenvironment. The photothermal effect enables the hydrogel to generate localized heat, further regulating wound healing (Priyadarshi et al., 2025). In wound healing, light-responsive hydrogels offer precise spatiotemporal control (Zhou et al., 2024), enabling the targeted initiation of drug release or modulation of cellular behavior in specific regions, minimizing the impact on healthy tissues. This capability overcomes the limitations of temperature-based stimuli and supports more frequent intervention (Ávila-Salas and Durán-Lara, 2020). Moreover, near-infrared (NIR) light, with its strong tissue penetration and low phototoxicity (Yan et al., 2016), combined with photothermal conversion materials (e.g., gold nanoparticles (Fatima et al., 2024), polydopamine nanoparticles, carbon-based nanomaterials), can effectively target deep wounds, such as deep burns or ulcers, through non-invasive remote control. Therefore, light-responsive hydrogels, with their precise control and non-invasive nature, offer new solutions for precise wound healing interventions, particularly in deep wound treatment and personalized medicine.

In addition to light-responsive hydrogels, hydrogels responsive to ultrasound, electric fields, magnetic fields, and pressure offer unique advantages and have become important components in the intelligent hydrogel toolbox. Ultrasound-responsive hydrogels utilize ultrasonic energy by incorporating sonosensitizers or microbubbles into the peptide network (Nele et al., 2020; Sun et al., 2022; Sirsi and Borden, 2014), producing cavitation effects (Coussios and Roy, 2008), localized heating, or ROS generation upon ultrasound irradiation, thereby regulating hydrogel swelling or drug release. The non-invasive nature of ultrasound, combined with its excellent tissue penetration, makes it a key tool for deep wound treatment, particularly for infection sites or tissue regeneration promotion (Sirsi and Borden, 2014; Chandan et al., 2020). Electric-responsive hydrogels, by incorporating conductive components (e.g., polypyrrole (Carayon et al., 2020), polyaniline, nanomaterials (Kolosnjaj-Tabi et al., 2019) or utilizing the inherent ion conductivity of peptides (Carayon et al., 2020), respond to external electric field stimulation by exhibiting rapid swelling, deswelling, osmotic pressure changes, or drug release (Carayon et al., 2020). This immediate, adjustable response is suitable for wound treatments requiring precise modulation of the electrophysiological microenvironment (Kolosnjaj-Tabi et al., 2019). Magnetic-responsive hydrogels incorporate superparamagnetic nanoparticles, such as Fe_3_O_4_ (Lee et al., 2019; Luo et al., 2010). Under an alternating magnetic field, these nanoparticles generate localized heat via the magnetothermal effect (Rittikulsittichai et al., 2016; Guo et al., 2005) or induce hydrogel deformation and directional migration through magnetic forces.Magnetic-responsive hydrogels are suitable for deep tissue interventions, effectively promoting angiogenesis or simulating the dynamic mechanical signals of the extracellular matrix (Shou et al., 2023). Pressure-responsive hydrogels, through specialized network structures or inherent swelling properties (He et al., 2023), respond to mechanical stress or changes in fluid pressure (Fang et al., 2020). During wound healing, pressure-responsive hydrogels can sense and adapt to dynamic changes in the wound site, providing adaptive management, reducing dressing change frequency, and improving patient comfort (Ning et al., 2025). A representative example is a multi-responsive system developed by Supparesk et al., comprising silicone-coated magnetic nanoparticles assembled with gold nanorods (Aurods). This hybrid system responds to temperature, infrared light, and magnetic fields, producing significant thermal and optical effects for applications like remote-controlled drug delivery and thermotherapy (Rittikulsittichai et al., 2016). These multi-responsive hybrid particles show considerable potential in remote-controlled drug delivery and thermotherapy.

In summary, ultrasound, electric, magnetic, and pressure-responsive peptide hydrogels complement light-responsive hydrogels, each showcasing distinct advantages. Ultrasound responsiveness is suitable for deep penetration, electric responsiveness provides precise electrophysiological control, magnetic responsiveness enables non-contact force/thermal manipulation, and pressure responsiveness offers dynamic adaptability. These multimodal hydrogels collectively advance the progress of intelligent wound management, providing more efficient, personalized treatment options for wound healing. In the future, they will demonstrate greater potential in the biomedical field, especially in the precise intervention of wound healing.

## 3 Conclusion and outlook

Responsive peptide hydrogels have demonstrated significant potential in the field of wound healing. By modulating the physicochemical properties of these hydrogels, intelligent materials can precisely respond to changes in the wound microenvironment, such as pH, ROS, MMPs, Glu, etc., enabling dynamic control of drug release, promoting cellular repair, inhibiting infection, and accelerating healing. As various responsive mechanisms, such as light, temperature, ultrasound, electric fields, and magnetic fields, are gradually integrated into these systems, the applications of responsive hydrogels in deep wound treatment and chronic wound repair are expanding. Furthermore, the role of responsive peptide hydrogels is steadily increasing in the treatment of inflammatory skin diseases, intradermal drug delivery, pulmonary delivery, bone and cartilage repair, prevention of tendon adhesion, cancer therapy, and cardiovascular diseases.

However, the application of this technology still faces certain challenges, including further optimization of hydrogel biocompatibility, biodegradability, and the precision of stimulus-response mechanisms. Future research will focus on multifunctional, intelligent, and tunable hydrogel systems, as well as their integration with modern therapeutic technologies, to achieve more precise and personalized wound management strategies. Through further material innovation and interdisciplinary research, responsive peptide hydrogels are expected to provide more efficient, minimally invasive, and adaptable wound treatment solutions for clinical applications.

## Abbreviations

ROS, reactive oxygen species; MMPs, matrix metalloproteinases; Glu, glucose; ECM, extracellular matrix; TIMPs, tissue inhibitors of metalloproteinases; PEG, polyethylene glycol; GelMA, gelatin methacryloyl; PBA, phenylboronic acid; GOx, glucose oxidase; ConA, Concanavalin A; UCST, Upper Critical Solution Temperature; LCST, Lower Critical Solution Temperature; PNIPAM, poly(N-isopropylacrylamide); NIR, near-infrared; UV, ultraviolet; IR, infrared.
